# Effects of Transcranial Alternating Current Stimulation and Neurofeedback on Alpha (EEG) Dynamics: A Review

**DOI:** 10.3389/fnhum.2021.628229

**Published:** 2021-07-08

**Authors:** Mária Orendáčová, Eugen Kvašňák

**Affiliations:** Department of Medical Biophysics and Medical Informatics, Third Faculty of Medicine, Charles University in Prague, Prague, Czechia

**Keywords:** transcranial alternating current stimulation, neurofeedback, behavioral benefits, alpha activity, brain plasticity, mechanisms, online and offline effects, factors influencing variability of responsiveness

## Abstract

Transcranial alternating current stimulation (tACS) and neurofeedback (NFB) are two different types of non-invasive neuromodulation techniques, which can modulate brain activity and improve brain functioning. In this review, we compared the current state of knowledge related to the mechanisms of tACS and NFB and their effects on electroencephalogram (EEG) activity (online period/stimulation period) and on aftereffects (offline period/post/stimulation period), including the duration of their persistence and potential behavioral benefits. Since alpha bandwidth has been broadly studied in NFB and in tACS research, the studies of NFB and tACS in modulating alpha bandwidth were selected for comparing the online and offline effects of these two neuromodulation techniques. The factors responsible for variability in the responsiveness of the modulated EEG activity by tACS and NFB were analyzed and compared too. Based on the current literature related to tACS and NFB, it can be concluded that tACS and NFB differ a lot in the mechanisms responsible for their effects on an online EEG activity but they possibly share the common universal mechanisms responsible for the induction of aftereffects in the targeted stimulated EEG band, namely Hebbian and homeostatic plasticity. Many studies of both neuromodulation techniques report the aftereffects connected to the behavioral benefits. The duration of persistence of aftereffects for NFB and tACS is comparable. In relation to the factors influencing responsiveness to tACS and NFB, significantly more types of factors were analyzed in the NFB studies compared to the tACS studies. Several common factors for both tACS and NFB have been already investigated. Based on these outcomes, we propose several new research directions regarding tACS and NFB.

## Introduction

In recent years, there has been a massive development in research dealing with transcranial alternating current stimulation (tACS) and neurofeedback (NFB), which both represent the non-invasive brain modulation methods capable of modulating electroencephalogram (EEG) activity (Legarda et al., 2011; Herrmann et al., 2013; Sitaram et al., 2017). While NFB works on the principle of self-regulation of endogenous EEG activity (Enriquez-Geppert et al., 2017; Ros et al., 2020), tACS is based on delivering external electric fields capable of interacting with an ongoing EEG activity (Liu et al., 2018; Vöröslakos et al., 2018). Despite the vast differences in mechanisms of action of tACS and NFB, according to the thorough research, both methods can successfully modulate various EEG bands (Lubar, 1997; Weber et al., 2011; Staufenbiel et al., 2014; Witkowski et al., 2016; Violante et al., 2017; Wischnewski and Schutter, 2017; Pimenta et al., 2018; Tseng et al., 2018; Abellaneda-Pérez et al., 2020). Both neuromodulatory methods have been also investigated in their ability to improve various brain functions such as motor performance (Joundi et al., 2012; Ros et al., 2014b; Scharnowski et al., 2015; Moisa et al., 2016; Guerra et al., 2018, 2019; Bologna et al., 2019), memory processes (Alexeeva et al., 2012; Polanía et al., 2012; Violante et al., 2017; Dobrakowski and Łebecka, 2020), attention (Escolano et al., 2014; Hopfinger et al., 2017; Berger and Davelaar, 2018; Deiber et al., 2020), creativity (Gruzelier, 2009; Agnoli et al., 2018; Di Bernardi Luft et al., 2018), emotional regulation (Johnston et al., 2010; Bramson et al., 2020), etc. (Hohn et al., 2019; Prim et al., 2019). These promising outcomes gave rise to new questions and new research directions regarding the link between EEG activity and brain functions (Enriquez-Geppert et al., 2017; Vosskuhl et al., 2018; Batail et al., 2019). Several studies of tACS and NFB examined immediate effects on an ongoing EEG activity (online effects) (Bazanova et al., 2009; Alagapan et al., 2016; Witkowski et al., 2016) while the other studies of tACS and NFB focused on studying the poststimulation (offline) EEG activity (Hanslmayr et al., 2005; Ros and Gruzelier, 2011; Neuling et al., 2013; Kasten et al., 2016).

In this review, we decided to compare tACS and NFB relative to the mechanisms of their effects on EEG activity, including the effects on the alpha band, the duration and the potential benefits of alpha-band aftereffects (offline alpha activity), and state-dependent factors, which may be responsible for variability in brain responsiveness to tACS and NFB. The alpha band, as a target EEG band of our interest, was deliberately selected since many studies of NFB and tACS focused on this EEG band, thereby making the alpha band to be a perfect candidate for this kind of comparative review. In the final part of this article, the conclusions about common and different findings for tACS and NFB are made and new research directions are proposed.

## Mechanisms Of tACS aND NFB

The effects of tACS and NFB on the targeted EEG activity can be studied in two ways: first of all, immediate (online) effects on an ongoing EEG activity can be investigated. Such a kind of investigation requires the intervention of tACS/NFB and simultaneous EEG recording (Karabanov et al., 2016; Neuling et al., 2017; Ros et al., 2020). Second, poststimulation EEG activity (offline activity/aftereffect) can be studied (Sitaram et al., 2017; Tavakoli and Yun, 2017; Batail et al., 2019). Both fields of research, tACS and NFB, include the studies dealing with the online effects as well as the offline effects of a particular neuromodulation technique on EEG activity (Fell et al., 2002; Hanslmayr et al., 2005; Ros et al., 2010; Helfrich et al., 2014b; Alagapan et al., 2016; Kasten et al., 2016). TACS and NFB scrutinized the possible mechanisms responsible for the induction and maintenance of both online and offline effects (Legenstein et al., 2008; Sitaram et al., 2017; Liu et al., 2018; Vöröslakos et al., 2018; Batail et al., 2019). The next section is dedicated to the mechanisms responsible for the online and offline effects of tACS and NFB.

### Mechanisms of tACS and NFB Responsible for Online Effects

Neurofeedback and tACS operate on completely different principles. EEG modulation *via* tACS is done for delivering external electric fields, which are capable of interacting with endogenous EEG activity (Antal and Paulus, 2013). For tACS, two or more scalp electrodes are required to soak in a conductive medium among which alternating current may pass (Antal and Paulus, 2013; Liu et al., 2018). The orientation of applied electric fields toward the stimulated regions is considered to play a crucial role in the effects of tACS on EEG activity (Neuling et al., 2012; Battleday et al., 2014; Hindriks et al., 2014). The orientation of the electric field to the stimulated region is essential since perpendicular and parallel orientations of the electric field to the particular brain areas lead to quantitatively different effects (Battleday et al., 2014; Liu et al., 2018).

In relation to the online effects of tACS, entrainment and intrinsic endogenous resonance have been considered to be the main mechanisms (Ali et al., 2013; Schmidt et al., 2014; Krause et al., 2019; Johnson et al., 2020). Entrainment refers to the phenomenon when the EEG activity having the same or very similar frequency to the tACS frequency becomes phase aligned to the external driving tACS frequency (Krause et al., 2019). Endogenous intrinsic resonance refers to the phenomenon when the frequency of an external driving force equals or is very similar to the dominant frequency of the stimulated system. When these two frequencies equal or are very similar to each other, low energy of an externally stimulating frequency is required to make the system to oscillate in its natural dominant frequency (Pikovsky et al., 2002). This physical phenomenon has been observed in the behavior of EEG activity when stimulated by tACS. In comparison to the other EEG frequencies, EEG having the same or very similar frequency to tACS showed the greatest increase in amplitude when stimulated by tACS (Ali et al., 2013; Schmidt et al., 2014). Nevertheless, there is still a gap of knowledge related to the precise mechanisms responsible for the effects of tACS on brain activity as current evidence speaking in favor of the aforementioned tACS mechanisms are rather indirect (Zaehle et al., 2010; Krause et al., 2019; Chen et al., 2021; Frohlich and Townsend, 2021).

In contrast to tACS, NFB-related modulation of EEG is based on endogenous self-regulation of brain activity using NFB-rewarded patterns of brain activity (Egner and Sterman, 2014; Wang et al., 2016; Othmer and Othmer, 2017). NFB includes several different modalities such as EEG biofeedback, functional MRI (fMRI), and functional near-red spectroscopy (fNIRS) (Muñoz-Moldes and Cleeremans, 2020). In this review, we are dealing with the EEG modality of NFB. EEG biofeedback works on the principle of providing the brain with information about its own functioning. For this purpose, EEG activity is detected from the electrodes on the participant's scalp. The target EEG activity, which is intended to be modulated, is set as the NFB-rewarded frequency (Egner and Sterman, 2014; Othmer and Othmer, 2017). Once the particular NFB-rewarded EEG activity reaches the level, which is at least as high as the rewarding threshold, the NFB system generates visual and/or auditory feedback (Enriquez-Geppert et al., 2017). The brain seems to have an innate ability to recognize and associate the rewarding feedback with its own activity, and it consequently becomes easier and easier for the brain to generate the EEG activity rewarded by NFB (Enriquez-Geppert et al., 2017; Sitaram et al., 2017; Melnikov, 2021). Within NFB research, there is still an ongoing debate whether NFB learning is an implicit or explicit type of learning or both (Enriquez-Geppert et al., 2017; Sitaram et al., 2017; Othmer, 2019; Muñoz-Moldes and Cleeremans, 2020; Melnikov, 2021). As well as in the case of tACS, studying direct evidence and the evolution of the putative mechanisms responsible for NFB learning represents a formidable challenge, and therefore the current research is mostly based on indirect correlations between NFB protocols and altered brain functioning (Melnikov, 2021; Olson et al., 2021).

In the realm of EEG biofeedback studies, a unidirectional and bidirectional training protocol is used (Dempster and Vernon, 2009; Friedrich et al., 2015). The unidirectional protocol refers to the NFB protocol aimed at modulating a single-frequency bandwidth (Dempster and Vernon, 2009; Weber et al., 2011). Unidirectional protocols can be single frequency (e.g., uptraining of alpha amplitude) or multifrequency (e.g., downregulation of beta and gamma amplitude) (Vanneste et al., 2016). Bidirectional protocols are multifrequency by definition, i.e., one frequency bandwidth is upregulated (e.g., the amplitude of sensorimotor rhythm up) with simultaneous downregulation of another frequency bandwidth (e.g., downregulation of the amplitude of theta bandwidth) (Dempster and Vernon, 2009; Friedrich et al., 2015; Enriquez-Geppert et al., 2017).

In contrast to tACS, which uses a single-frequency stimulation (e.g., 10 Hz for alpha-band stimulation), NFB protocols usually train the whole bandwidth (e.g., the whole alpha bandwidth 8–13 Hz up). In multifrequency protocols, the NFB research field contains many studies using multifrequency protocols (Peniston and Kulkosky, 1991; Egner et al., 2002; Dohrmann et al., 2007; Friedrich et al., 2015; Vanneste et al., 2016; Güntensperger et al., 2019) whereas the multifrequency protocols in tACS seem to be quite an emerging field of research with a much shorter history than multifrequency NFB protocols (Helfrich et al., 2016; Lara et al., 2018; Bramson et al., 2020; Turi et al., 2020).

To make the outcomes of tACS and NFB studies as comparable as possible, we decided to compare single- and multi-session alpha tACS studies with single- and multi-session unidirectional alpha NFB protocols. Multifrequency tACS and NFB studies were excluded from the analysis.

The following chapter is devoted to scrutinizing the online effects of tACS and NFB on alpha-band activity.

### Online Effects of tACS and NFB on Alpha Band

An investigation of the online effects of tACS represents a formidable challenge since massive artifacts occur when electrical stimulation is applied (Minami S, 2017; Neuling et al., 2017; Kasten and Herrmann, 2019). The occurrence of tACS-induced electric artifacts causes great interpretation pitfalls in the analysis of the effects of tACS on online EEG activity as tACS-induced electric artifacts occur in the same or very similar frequency as EEG frequency of our interest (Neuling et al., 2017; Kasten et al., 2018a; Kasten and Herrmann, 2019) and/or in its harmonics Minami S, 2017; Kasten et al., 2018b). The magnitude of these artifacts is several orders larger than the magnitude of an EEG signal of our interest (Kasten and Herrmann, 2019). Therefore, most tACS studies have focused on the evaluation of the offline effects of tACS rather than on evaluating the online ones (Zaehle et al., 2010; Neuling et al., 2013; Vossen et al., 2015; Kasten et al., 2016; Stecher et al., 2017; Prim et al., 2019). However, there are a couple of tACS studies, which managed at least to partially overcome that problem and the measured effects of tACS on an ongoing EEG activity (Helfrich et al., 2014b; Ruhnau et al., 2016; Kasten et al., 2018a; Castellano et al., 2020). For an analysis of the online EEG activity, the interleaved EEG-tACS protocol uses very short intervals of tACS, which are separated by short non-stimulation periods during which EEG activity is recorded (Castellano et al., 2020). This kind of measurement was exploited in Castellano et al. (2020) study, which reported that 10 Hz tACS was delivered for 27 min among PO3, PO4, and Oz in the following way: 5-s tACS epochs were separated by epochs during which EEG was recorded. A particular study showed increases in alpha amplitude after 5-s intervals of 10-Hz tACS (Castellano et al., 2020).

Also, EEG can be monitored during continuous tACS, and tACS-induced artifacts are removed in poststimulation EEG analysis *via* the exploitation of artifact-rejection technique (Helfrich et al., 2014a; Ruhnau et al., 2016; Witkowski et al., 2016). Due to this method, the alterations in alpha activity during tACS were studied in some alpha tACS studies (Helfrich et al., 2014b; Ruhnau et al., 2016). Increased phase-locking in the online alpha band has been observed in one tACS study in which 10 Hz tACS was applied for 20 min to Oz–Cz regions (Helfrich et al., 2014b). Another tACS study found increased phase coherence in online alpha during 0.65 mA tACS to Oz–Cz regions. Interestingly, a significant increase in alpha-phase coherence was observed only during the eyes-open condition (2 min) and not during the eyes-closed condition (2 min) (Ruhnau et al., 2016). However, complete rejection of tACS-induced artifacts from EEG recordings is impossible (Noury et al., 2016; Kasten et al., 2018b; Kasten and Herrmann, 2019). Although the aforementioned artifact-rejection technique is capable of a significant reduction of tACS-induced artifacts having the stationary amplitude, it is incapable of removing nonstationary artifacts with amplitude fluctuating over time (Noury et al., 2016; Kasten and Herrmann, 2019). Such non-stationary artifacts result from the physiological changes, such as heartbeats and respiratory movements (Noury et al., 2016), which have been found to modulate the amplitude of electric artifacts induced by tACS itself (Noury et al., 2016; Kasten and Herrmann, 2019). The mechanisms responsible for this kind of amplitude modulation of tACS-induced artifacts in EEG have been proposed to have their origin in changes in skin conductance whereas the head movements are thought to modulate the amplitude of tACS-induced artifacts in magnetoencephalography (MEG) (Kasten and Herrmann, 2019). Apart from the aforementioned artifact-rejection technique, two other methods have been used to minimize tACS-induced artifacts in EEG/MEG. First, intracranial spikes were monitored in animal studies (Krause et al., 2019). Since there is a great difference between the morphology of a tACS waveform and the morphology of waveforms of the investigated neuronal activity, the smaller influence of tACS-induced artifact was thought to contaminate EEG data compared to the situations in which a tACS waveform and a waveform of the investigated neuronal populations would be the same (Krause et al., 2019). However, the great disadvantage of this kind of intracranial measurement is that it cannot be applied in human tACS studies due to the ethical issues (Krause et al., 2019). Another way of minimizing the tACS artifact partially resembles the previous one as it also consists in creating the difference between the waveform morphology of tACS and the waveform morphology of the stimulated EEG activity (Dowsett and Herrmann, 2016). Its underlying principle is based on using a different tACS waveform than the stimulated EEG waveform (Dowsett and Herrmann, 2016). The exploitation of this method led to a significant reduction of tACS-induced artifacts having the same frequency as the studied EEG activity, but it also led to an induction of massive artifacts in the harmonics of target EEG frequency (Dowsett and Herrmann, 2016). Taken together, it seems that there is no way of the complete elimination of tACS-induced artifacts from EEG recordings, and therefore it is necessary to interpret the online effects of tACS on EEG activity with great caution (Dowsett and Herrmann, 2016; Minami S, 2017; Kasten and Herrmann, 2019).

In contrast to tACS, NFB does not produce a significant EEG artifact as such, so the analysis of online EEG during NFB intervention is easier. There are also many NFB studies, which investigated the effects of alpha NFB on online alpha activity (Fell et al., 2002; Bazanova et al., 2009; Ibric et al., 2009; Ros et al., 2010; Ros and Gruzelier, 2011). A progressive increase in alpha amplitude was observed in alpha-amplitude upregulated NFB protocols (Fell et al., 2002; Bazanova and Shtark, 2007; Bazanova et al., 2009; Ros et al., 2010). The greatest amplitude increase was often seen in the final minutes of NFB training (Fell et al., 2002; Ros et al., 2010), which is in accordance with the prediction that NFB-conditioned neuronal networks require some time to learn and adopt the appropriate NFB strategy to upregulate the rewarded neural activity (Legenstein et al., 2008; Sitaram et al., 2017).

As with tACS, alpha coherence was found to be modulated by NFB protocols (Bazanova et al., 2009; Ibric et al., 2009; Mottaz et al., 2015). Coherence was found to be decreased and increased in the coherence downregulation protocol (Ibric et al., 2009) and in the coherence upregulation protocol (Mottaz et al., 2015), respectively. Alpha coherence was also found to be increased after the alpha-amplitude upregulation protocol (Bazanova et al., 2009).

Alpha-band frequency bandwidth was reported to be influenced in tACS research (Helfrich et al., 2014b) and also in NFB studies (Fell et al., 2002; Bazanova and Aftanas, 2010). Alpha tACS was found to lead to a sharpening of the peak frequency, i.e., narrowing of the alpha frequency band around the alpha frequency value that equaled or was very close to the tACS frequency (Helfrich et al., 2014b). This phenomenon was also reported within NFB research after using the alpha-amplitude upregulation protocol (8–13 Hz) (Fell et al., 2002). A different trend was observed in one NFB study that reports the comparison of a fixed alpha-band (8–13 Hz) protocol with an individualized alpha-band protocol aimed at upregulating alpha amplitude (Bazanova and Aftanas, 2010). This study showed that a fixed alpha-band protocol led to a narrowing of the alpha band plus it has led to rigidity in event-related synchronization (ERS) and event-related desynchronization (ERD) in the alpha band. These EEG changes were connected with a worsening of behavioral symptoms. On the contrary, using the individualized alpha-band protocol resulted in a broadening of the alpha band accompanied by greater depths of ERD and ERS in the alpha band, which went hand in hand with improvements in many behavioral domains (Bazanova and Aftanas, 2010).

Alpha modulation by tACS and NFB was shown to influence other EEG frequencies too. Some types of cross-frequency interaction (CFI) were found within both tACS and NFB research (Hanslmayr et al., 2005; Nan et al., 2012; Helfrich et al., 2016; Herring et al., 2019). Alpha–gamma CFI has been frequently reported during alpha tACS (Helfrich et al., 2016; Herring et al., 2019; Castellano et al., 2020). Alpha–gamma phase-amplitude CFI was observed during 20 min of 1 mA 10 Hz tACS applied to Oz–Cz regions (Helfrich et al., 2016). A different kind of CFI was reported in Castellano et al. (2020) study in which amplitudes of both alpha and gamma bands were increased during 20 min of 10 Hz tACS passing among PO3, PO4, and Oz (Castellano et al., 2020). On the contrary, another study found that gamma power was decreased during tACS, with the individualized intensity and individualized alpha frequency, passing between Oz and Cz (Herring et al., 2019). Similarly, alpha–gamma antagonism was reported in NFB studies as well (Ros et al., 2010; Bagherzadeh et al., 2020). Interestingly, many tACS and NFB studies found a great consistency in the following alpha–gamma CFI tendency, i.e., the upregulation of alpha activity is accompanied by the downregulation of gamma activity and *vice versa* (Ros et al., 2010; Helfrich et al., 2014b, 2016; Bagherzadeh et al., 2020). Such outcomes seem to be in accordance with the previous findings discovering the antagonistic relationship between alpha and gamma in their functions relative to the regulation of overall arousal (Spaak et al., 2012; Herrera et al., 2016).

### Mechanisms of tACS and NFB Responsible for Offline Effects

Apart from the online effects of tACS and NFB, both research fields report a considerable number of studies reporting massive poststimulation changes in alpha activity following alpha NFB (Hanslmayr et al., 2005; Ros et al., 2010; Nan et al., 2012; Lavy et al., 2019) and tACS (Veniero et al., 2015; Kasten et al., 2016; Prim et al., 2019). Poststimulation brain activity in the stimulated EEG band is termed as “offline” or “aftereffect” (Veniero et al., 2015; Krause et al., 2019; Bagherzadeh et al., 2020). Both NFB and tACS have been found to modulate amplitude (Bazanova et al., 2009; Ros et al., 2010; Kasten and Herrmann, 2017; Wischnewski and Schutter, 2017), coherence (Bazanova et al., 2009; Neuling et al., 2013; Mottaz et al., 2015; Stonkus et al., 2016; Kasten and Herrmann, 2017; Schubert et al., 2020), frequency (Ahn et al., 2019; Lavy et al., 2019), and CFIs (Nan et al., 2012; Helfrich et al., 2016; Vanneste et al., 2016).

A possible mechanism responsible for inducing and maintaining aftereffects in the stimulated EEG is brain plasticity. It has been proposed to play a major role in the mechanisms of tACS and NFB (Legenstein et al., 2008; Zaehle et al., 2010; Ros et al., 2014a; Sitaram et al., 2017; Wischnewski and Schutter, 2017; Batail et al., 2019).

The effects of tACS or NFB on brain plasticity have been already investigated by using fMRI (Kluetsch et al., 2014; Nicholson et al., 2016; Abellaneda-Pérez et al., 2020; Gundlach et al., 2020; Mondino et al., 2020; Chen et al., 2021), the measurement of a motor-evoked potential (MEP) by transcranial magnetic stimulation (TMS) (Ros and Gruzelier, 2011; Wach et al., 2013; Vallence et al., 2021). The link between EEG changes and changes in the levels of the concentration of the molecular substrates of plasticity after tACS/NFB intervention has been already studied too Lee et al., 2019; Wischnewski et al., 2019; Markiewicz and Dobrowolska, 2020; Riddle et al., 2020a). For instance, Wischnewski et al. (2019) discovered the application of dextromethorphan, which is an N-Methyl-D-aspartate (NMDA) receptor antagonist (Ferkany et al., 1988) completely erased any tACS-induced aftereffects on beta-band activity. This effect was not present in the control group, which did not receive dextromethorphan (Wischnewski et al., 2019). In the control group, the aftereffects persisted for ~20 min after tACS (Wischnewski et al., 2019), which is quite comparable with the aftereffects' durations of other tACS studies, which entrained other EEG bands (Neuling et al., 2013; Helfrich et al., 2014a; Berger et al., 2018). NMDA receptor activity is heavily involved in Hebbian plasticity (Cotman et al., 1988; Rauschecker, 1991). The study by Wischnewski et al. (2019) therefore managed to provide unique evidence of a causal link between tACS-induced aftereffects and Hebbian plasticity (Wischnewski et al., 2019). Another tACS work investigated whether a causal relationship exists between different alleles of the gene coding for a brain-derived neurotrophic factor (BDNF) and alpha-band responses to alpha tACS (Riddle et al., 2020a). BDNF, which is involved in the regulation of Hebbian as well as homeostatic plasticity (Turrigiano and Nelson, 2000), is coded by at least two different alleles, i.e., Val666Met and Val666Val (Katerberg et al., 2009). In a study by Riddle et al. (2020a), a DNA analysis was done on saliva taken from the participants in three independent alpha tACS studies. The alpha amplitudes of the participants with Val666Val exhibited significantly smaller amplitude responses to alpha tACS than the participants with Vall666Met (Riddle et al., 2020a). On the other hand, another tACS study did not find any differences between the magnitude of responsiveness to tACS and BDNF different alleles (Guerra et al., 2020). In the same vein, BDNF has been theorized to be linked with greater magnitudes of EEG responses to NFB (Markiewicz et al., 2017), which was later experimentally tested by measuring the level of concentration of BDNF before and after multi-session NFB intervention (Lee et al., 2019; Markiewicz and Dobrowolska, 2020). While one study found a remarkable upregulation of BDNF in the post-NFB period compared to the pre-NFB period (Markiewicz and Dobrowolska, 2020), the second study did not find any significant differences between the pre-NFB period and the post-NFB period (Lee et al., 2019).

Taken together, both tACS and NFB fields of research involve the studies investigating a potential link between aftereffects and the structural and functional correlates of neuroplasticity.

## Aftereffects

Aftereffects induced by non-invasive neuromodulation techniques represent a broadly investigated domain of interest in both tACS and NFB fields of research in relation to their dynamics, duration, and behavioral benefits (Zaehle et al., 2010; Zoefel et al., 2011; Veniero et al., 2015; Kasten and Herrmann, 2017; Sitaram et al., 2017; Batail et al., 2019; Haberbosch et al., 2019; Mondino et al., 2020; Vallence et al., 2021). In this section, alpha aftereffects induced by tACS and NFB are compared regarding their dynamics, duration, behavioral benefits, and their potential link with brain plasticity. Single-session tACS and NFB as well as multi-session studies will be discussed.

### Dynamics

Aftereffects dynamics related to EEG properties (e.g., amplitude and coherence) have been already studied in both single- and multi-session tACS and NFB studies (Ros et al., 2010; Zaehle et al., 2010; Neuling et al., 2013; He et al., 2019).

#### Single-Session Studies

**Figure 1:**
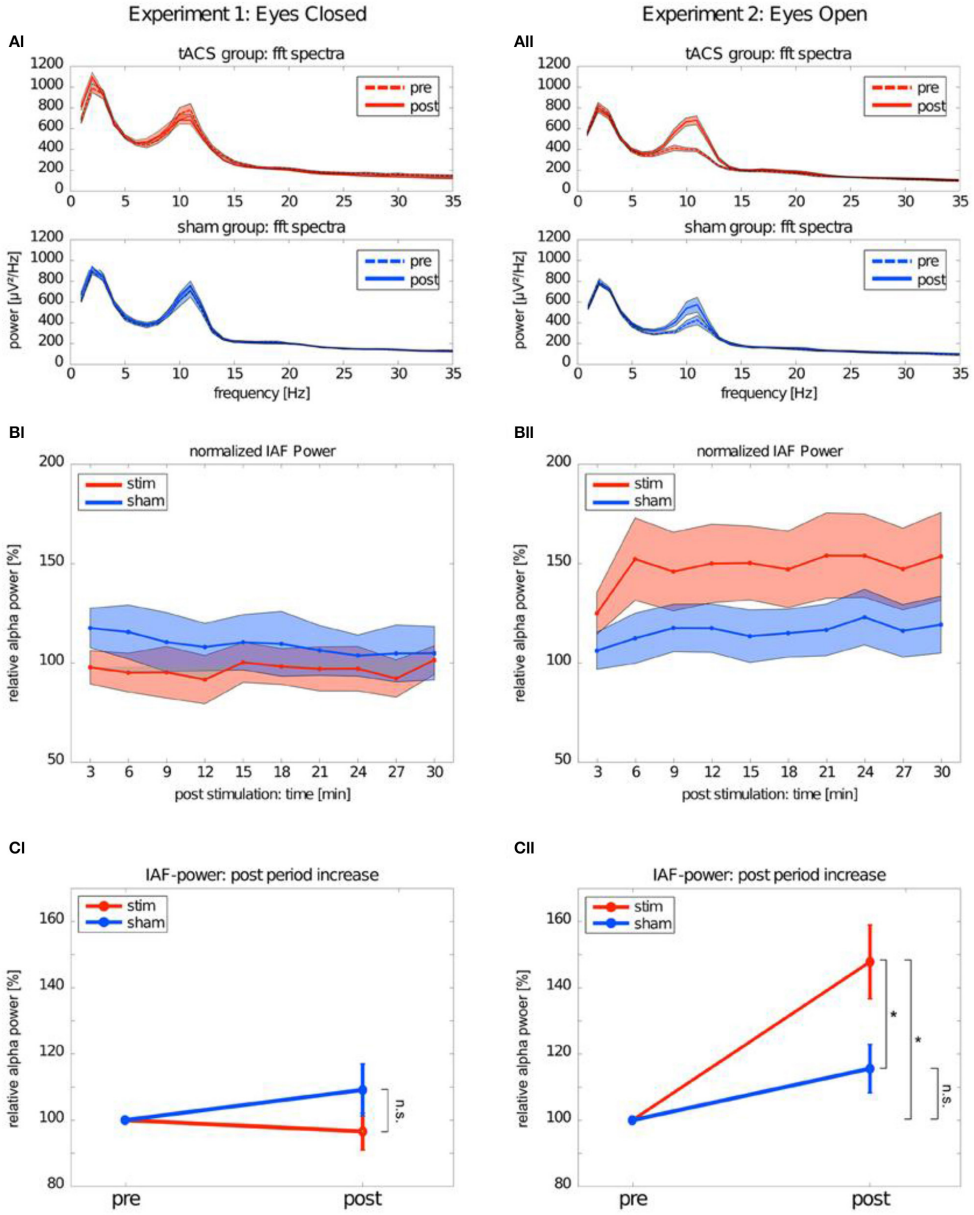
Alpha band after transcranial alternating current stimulation (tACS). This figure depicts the difference in EEG power spectrum between Experiment 1: Eyes-closed and Experiment 2: Eyes-open conditions. **(AI)** tACS group:fft spectra depicts pre- and post-stimulation changes in power spectrum for EEG frequencies in real tACS in eyes-closed condition. Axis *y* stays for power values (microvolts) and axis *x* stands for frequency values (Hz). tACS group: fft spectra depicts pre- and post-stimulation changes in power spectrum for EEG frequencies in sham tACS in eyes-closed condition. **(BI)** Normalized IAF power represents pre- and post-stimulation difference in relative power of individual alpha frequency (IAF) in real tACS group in eyes-closed condition. Axis *y* stays for power values (microvolts) and axis *x* stands for frequency values (Hz). **(CI)** IAF power post period increase represents pre- and post-stimulation difference in relative alpha power between stim and sham in eyes-closed condition. **(AII)** tACS group:fft spectra depicts pre- and post-stimulation changes in power spectrum for EEG frequencies in real tACS in eyes-open condition. Axis *y* stays for power values (microvolts) and axis *x* stands for frequency values (Hz). tACS group: fft spectra depicts pre- and post-stimulation changes in power spectrum for EEG frequencies in sham tACS in eyes-open condition. **(BII)** Normalized IAF power represents pre- and post-stimulation diference in relative power of individual alpha frequency (IAF) in real tACS group in eyes-open condition. Axis *y* stays for power values (microvolts) and axis *x* stands for frequency values (Hz). **(CII)** IAF power post period increase represents pre- and post-stimulation difference in relative alpha power between stim and sham in eyes-open condition. The symbol *represents the diference in post-stimulation alpha power between stim and sham group in eyes-open condition. [with the author's permission, taken from Neuling et al. (2013).

Many single-session tACS studies focused on studying the poststimulation alpha-band changes report significant alpha aftereffects following tACS (Zaehle et al., 2010; Neuling et al., 2013; Wach et al., 2013; Helfrich et al., 2014b; Alagapan et al., 2016; Kasten et al., 2016; Kasten and Herrmann, 2017; Stecher et al., 2017; Berger et al., 2018). Increased alpha amplitude was observed in the studies focusing on comparing the alpha dynamics immediately after tACS with the alpha dynamics after longer elapsed times (minutes) following alpha tACS (Zaehle et al., 2010; Neuling et al., 2013; Kasten et al., 2016; Kasten and Herrmann, 2017; Stecher et al., 2017; Berger et al., 2018). Figure 1 depicts increase of alpha amplitude after alpha tACS. In line with the research, single-session NFB studies approached investigations of the aftereffect in alpha activity in the same fashion, i.e., comparing alpha dynamics immediately after NFB and after a longer elapsed time (Hanslmayr et al., 2005; Bazanova et al., 2009; Ros et al., 2010, 2013; Escolano et al., 2012). Increased alpha activity immediately after NFB and a longer elapsed time after NFB intervention has been repeatedly found for alpha amplitude and coherence with several types of alpha protocols: (1) alpha-amplitude upregulation protocols (Hanslmayr et al., 2005; Escolano et al., 2012), (2) alpha-amplitude downregulation protocols (Ros et al., 2010, 2013), (3) coherence upregulation protocols (Mottaz et al., 2015), and (4) coherence downregulation protocols (Ibric et al., 2009).

#### Multi-Session Studies

There are two possible ways of studying alpha-band modulation relative to multi-session interventions (Ros et al., 2020). The dynamics of alpha responses can be investigated during offline and online periods within multiple sessions of brain stimulation (Dempster and Vernon, 2009; Ros et al., 2020). Studying stimulation-induced offline alpha is done by including the measurements and consequent evaluation of EEG dynamics in the “silent” or no-stimulation period (Dempster and Vernon, 2009; Ros et al., 2020). Such measurements and comparisons can be made between the first and the last tACS session (Clancy et al., 2018; Ahn et al., 2019; He et al., 2019) and between the first and the last NFB session (Van Boxtel et al., 2012; Guez et al., 2015). Based on the current literature, the outcomes of both tACS (Ahn et al., 2019; He et al., 2019) and NFB studies (Van Boxtel et al., 2012) showed a significantly increased alpha activity during the offline period following the last session compared to the first session.

However, if the measurements are done only for the first and for the last sessions, less is known about the progressive session-to-session responsiveness dynamics of the alpha band to a particular neuromodulation method. To uncover session-to-session changes in the alpha-band dynamics, the behavior of the alpha band during the offline period can be measured and compared after and/or before each of the subsequent stimulation periods. This kind of measurement has been done in both tACS (Schmidt et al., 2013) and NFB studies (Dempster and Vernon, 2009; Kerson et al., 2009; Nan et al., 2012; Hsueh et al., 2016). A progressive session-to-session increase in offline alpha amplitude has been found in both tACS (Schmidt et al., 2013) and NFB studies (Cho et al., 2008; Zoefel et al., 2011).

The second type of evaluation of alpha dynamics investigates session-to-session changes in online alpha during the intervention period of tACS or NFB. In alpha-band NFB research, there are several studies, which have reported progressive session-to-session increases in alpha-band responsiveness to the NFB protocol. In the alpha-amplitude upregulation protocol, progressive session-to-session increases in alpha amplitude during the NFB sessions were reported in several studies (Zoefel et al., 2011; López-Larraz et al., 2012; Nan et al., 2013; Wan et al., 2014; Bobby and Prakash, 2017; Naas et al., 2019). Progressive increases in the ability to manipulate the alpha-band dynamics relative to the NFB protocol have been also observed in alpha-downregulation protocols (Wan et al., 2016; Nan et al., 2018, 2020). To the best of our knowledge, no study has investigated session-to-session online changes in the alpha-band responsiveness relative to multiple sessions of alpha tACS.

We did not find any multi-session alpha tACS studies investigating session-to-session alpha-band dynamics during tACS. In other words, all multi-session tACS were focused on the offline alpha activity (Schmidt et al., 2013; Clancy et al., 2018; Ahn et al., 2019; He et al., 2019). On the other hand, NFB research, which seems to have a longer history of studying the dynamics of alpha activity, involves a considerable number of multi-session studies focusing on studying the offline alpha (Kerson et al., 2009; Breteler et al., 2010; Alexeeva et al., 2012) as well as the online dynamics of alpha activity (Bobby and Prakash, 2017) or both (Plotkin, 1978; Dempster and Vernon, 2009; Escolano et al., 2011; López-Larraz et al., 2012; Nan et al., 2013, 2020; Dekker et al., 2014; Hsueh et al., 2016; Wan et al., 2016). With regard to the NFB studies devoted to showing the alpha behavior during both online and offline periods of the NFB session, it is worth mentioning that some studies reported an increase of alpha-band responsiveness relative to NFB modulation only during online periods and with no changes during offline periods (Nan et al., 2012, 2020; Hsueh et al., 2016; Wan et al., 2016). Conversely, several NFB studies reported the opposite, i.e., session-to-session increases in alpha-band responsiveness in offline periods but not during online ones (Cho et al., 2008; Dempster and Vernon, 2009).

Finally, several studies found progressive increases in alpha activity in line with the NFB protocol in both online and offline periods of multi-session interventions (Escolano et al., 2011; Zoefel et al., 2011; Dekker et al., 2014; Mottaz et al., 2015).

Apart from the changes in the coherence and the amplitude, also changes in the frequency and CFI are sometimes reported by multi-session tACS and NFB studies (Alexeeva et al., 2012; Nan et al., 2012; Ahn et al., 2019; Lavy et al., 2019). In one study, five consecutive 10 Hz tACS sessions led to a notable peak frequency sharpening of alpha frequency around 10 Hz bandwidth. These frequency-related changes have persisted approximately for 1 month (Ahn et al., 2019). A different kind of frequency modulation has been observed in one NFB study in which alpha-amplitude upregulation protocol led to a progressive increase of peak alpha-frequency activity (Alexeeva et al., 2012; Lavy et al., 2019). Alpha-amplitude upregulation protocol has also been shown to have a long-term effect on other frequency bandwidths (Nan et al., 2012). In Nan et al. (2012) study, session-to-session increase of alpha as well as low beta and simultaneous decrease of delta amplitude were observed in multi-sessions of alpha upregulation protocol (Nan et al., 2012). To the best of our knowledge, no multi-session tACS study has found/paid attention to the changes in other EEG frequencies yet after alpha tACS.

### Duration

The duration of aftereffects has been already studied by both single- and multi-session tACS and NFB studies (Zaehle et al., 2010; Ros and Gruzelier, 2011; Ghasemian et al., 2016; Ahn et al., 2019).

#### Single-Session Studies

Among the reviewed single-session studies, there are several tACS and NFB studies, which were dedicated to investigate the duration of electrophysiological and behavioral tACS- and NFB-induced aftereffects in the alpha band (Ros et al., 2010, 2013; Zaehle et al., 2010; Neuling et al., 2013; Kasten et al., 2016; Kasten and Herrmann, 2017; Stecher et al., 2017; Berger et al., 2018). In relation to the capability of tACS and NFB to induce long-lasting aftereffects in the target EEG bandwidth, the duration of the intervention period was found to play an important role (Fetz and Baker, 1973; Strüber et al., 2015; Cabral-calderin et al., 2016). It was shown that 1 s (Strüber et al., 2015) as well as 3 s of alpha tACS (Cabral-calderin et al., 2016) were too short periods to induce effects in the stimulated alpha bandwidth (Strüber et al., 2015; Cabral-calderin et al., 2016). Similarly, the establishment of strategies leading to a successful NFB training does not occur immediately after starting of NFB training, but it occurs after several minutes after starting the NFB session (Fetz and Baker, 1973; Fell et al., 2002; Bazanova et al., 2009; Ros et al., 2010). Durations of single-session tACS interventions reporting long-lasting electrophysiological aftereffects (i.e., alterations in amplitude and/or coherence in the alpha bandwidth, or changes in the MEP amplitude) ranged from 8 to 20 min and the reported durations of alpha-band aftereffects ranged from 3 to 70 min. Table 1 gives a more detailed overview of the duration of tACS interventions and the duration of the consequent alpha-band aftereffects.

**Table 1:** Duration of transcranial alternating current stimulation (tACS) interventions and alpha-band electrophysiological aftereffects.

**References**	**Intervention duration**	**Aftereffect duration**
Zaehle et al. (2010)	10 min	At least 3 min
Stecher et al. (2017)	8 min	10 min
Wach et al. (2013)	10 min	30 min
Neuling et al. (2013)	20 min	At least 30 min
Berger et al. (2018)	20 min	30 min
Kasten and Herrmann (2017)	20 min	At least 30 min
Kasten et al. (2016)	20 min	70 min

Comparable outcomes have been reported in NFB studies showing that 30 min of NFB training resulted in 20–30 min of aftereffects (Ros et al., 2010, 2013; Ros and Gruzelier, 2011). In relation to the behavioral aftereffects associated with tACS and NFB, 20–30 min of both tACS and NFB have been demonstrated to lead to long-term behavioral aftereffects (Ghasemian et al., 2016; Kasten and Herrmann, 2017) lasting for at least 50 min after tACS (Kasten and Herrmann, 2017) and 90 min after NFB (Ghasemian et al., 2016). Based on the findings reported above, a single tACS or NFB intervention lasting 20–30 min seems to induce aftereffects in the alpha bandwidth with a comparable duration of persistence.

#### Multi-Session Studies

Some multi-session NFB and tACS studies have also investigated the potential duration of aftereffects in offline alpha (Kerson et al., 2009; Alexeeva et al., 2012; Van Boxtel et al., 2012; Mottaz et al., 2015; Ahn et al., 2019).

Changes in alpha amplitude lasting approximately for 1 month were seen after 5 consecutive days of alpha tACS (Ahn et al., 2019; Alexander et al., 2019). Similarly, long-term aftereffects following the multi-session alpha NFB training have been reported by NFB studies (Kerson et al., 2009; Alexeeva et al., 2012; Van Boxtel et al., 2012; Mottaz et al., 2015). In relation to both electrophysiological and behavioral aftereffects, the long-term alpha-band aftereffects have been reported. For example, 15 sessions of the NFB alpha-amplitude upregulating protocol led to a significant increase in alpha amplitude, which persisted for at least 3 months (Van Boxtel et al., 2012). Comparable duration of persistence of aftereffects after alpha-band training was observed after seven sessions of the alpha coherence up-training protocol, leading to behavioral improvements, which have persisted for at least 6 weeks (Mottaz et al., 2015). Another NFB study reported electrophysiological and behavioral aftereffects that have lasted for at least 1 month after the termination of 11 NFB sessions (Alexeeva et al., 2012). Another NFB study focused on the measurements of electrophysiological and behavioral aftereffects using completely different recording time periods (Kerson et al., 2009). The evaluation of electrophysiological aftereffects using EEG recordings revealed that significant and sustained aftereffects were present only for 1 week after the completion of all experimental sessions. However, the behavioral assessment that was done 6 months after the termination of the whole experiment showed that behavioral improvement was still present (Kerson et al., 2009). Comparably, 6 months of improved behavioral functioning was found after 5 consecutive days of alpha tACS (Riddle et al., 2020b).

All in all, both single- and multi-session tACS and NFB studies are capable of inducing long-term aftereffects with a comparable duration of persistence. Nevertheless, in comparison to NFB, there are not so many multi-session tACS studies investigating behavioral and electrophysiological aftereffects. For that reason, in order to determine the similarities/differences in qualitative and quantitative aspects of aftereffects induced by tACS and NFB, future studies dedicated to systematic investigation of this issue are required.

### Behavioral Benefits

Behavioral improvements associated with aftereffects have been broadly investigated by both tACS and NFB studies (Hanslmayr et al., 2005; Bazanova et al., 2009; Kerson et al., 2009; Kasten and Herrmann, 2017; Berger and Davelaar, 2018; Ahn et al., 2019; Alexander et al., 2019; Deiber et al., 2020).

#### Single-Session Studies

Several single-session studies have reported a positive correlation between improved neurophysiological functioning and the magnitude of the aftereffects in tACS (Kasten and Herrmann, 2017; Prim et al., 2019) and NFB fields of research (Hanslmayr et al., 2005; Bazanova and Shtark, 2007; Bazanova et al., 2009; Escolano et al., 2012). The beneficial influence of increased alpha amplitude in the alpha-amplitude uptraining protocol was demonstrated in musical performance, which was notably improved after 20 min of individual NFB alpha-amplitude upregulation (Bazanova and Shtark, 2007). Musical improvement went hand in hand with NFB-induced elevation of alpha amplitude (Bazanova and Shtark, 2007). Another promising finding revealed a positive link between improved psychomotor performance and increased post-training alpha amplitude after 30 min of upregulating individual upper alpha amplitude in the fronto-occipital areas (Bazanova et al., 2009). In relation to the beneficial effects of alpha aftereffects after tACS, one study documented its beneficial effects of increased alpha amplitude on the level of cognitive performance (Kasten and Herrmann, 2017). In that study, 20 min of individual alpha (IAF) tACS led to a notable improvement in subsequent mental task performance (Kasten and Herrmann, 2017). Compared to the baseline alpha level, poststimulation alpha levels were notably increased immediately after tACS and also in the pre-stimulus (resting) period during subsequent cognitive performance. A positive correlation between increased cognitive performance in pre-stimulus alpha power was found during the assessment of a cognitive task after tACS (Kasten and Herrmann, 2017). Intriguingly, the same type of cognitive task was studied in NFB studies. These NFB studies also reported a positive correlation between increased pre-stimulus alpha levels and improved mental performance 25–30 min after NFB training (Hanslmayr et al., 2005; Escolano et al., 2012). Similarly, as with the findings of Kasten and Herrmann (2017), NFB studies found an increase in alpha amplitude immediately after the intervention period as well as increased alpha amplitude in the pre-stimulus period of subsequent cognitive performance (Hanslmayr et al., 2005; Escolano et al., 2012). In sum, both alpha tACS and NFB studies report positive correlations between the magnitude of the alpha aftereffect and the level of behavioral improvements.

#### Multi-Session Studies

Correlations between the magnitude of alpha aftereffect and the level of behavioral performance have been studied by both tACS and NFB multi-session studies (Zoefel et al., 2011; Berger and Davelaar, 2018; Clancy et al., 2018; Ahn et al., 2019).

Starting with tACS studies, the beneficial effects of alpha modulation have been studied in the connection with the improvement in schizophrenia-related symptoms (Ahn et al., 2019). Schizophrenia-related symptoms were found to be accompanied by a reduced alpha activity (Omori et al., 1995; Hong et al., 2008). In a study by Ahn et al. (2019), 10-Hz tACS using 1 mA was applied to the fronto-temporal network twice a day for 5 consecutive days (Ahn et al., 2019). After 5 days of tACS, significant reductions in schizophrenia-related auditory hallucinations as well as a notable increase in offline alpha activity were observed. Significant correlations were also found between the magnitude of improvement of clinical symptoms and the elevation of alpha amplitude (Ahn et al., 2019). Another multi-session alpha tACS study focused on examining the influence of multiple alpha tACS on the modulation of anxiety levels (Clancy et al., 2018). Clancy et al. (2018) applied 2-mA alpha tACS for 4 consecutive days. Interestingly, even after the first tACS session, there was a reduction in anxious arousal and an increase in the perception of pleasant stimuli, which has persisted for at least 24 h, i.e., to the beginning of the next tACS session (Clancy et al., 2018). After the completion of all four tACS sessions, a remarkable reduction in anxiety was observed and the level of clinical improvement was positively associated with the magnitude of the tACS-induced increase in offline alpha activity (Clancy et al., 2018). The findings also agree with NFB studies, which managed to reduce anxiety by enhancing alpha amplitude (Hardt and Kamiya, 1978; Dadashi et al., 2015). Apart from a reduction in anxiety, several other promising clinical outcomes, such as an improvement in spelling in dyslectic patients (Breteler et al., 2010), clinical improvement in post-stroke patients (Mottaz et al., 2015), working memory improvement (Zoefel et al., 2011), and the improvement of visual performance, have been reported (Nan et al., 2013).

There are also NFB studies reporting positive correlations between the level of behavioral improvement and the magnitude of increased online alpha activity (Nan et al., 2012, 2013; Bobby and Prakash, 2017). A positive correlation between the magnitude of behavioral improvement and the magnitude of online alpha-band responsiveness to NFB has been found for working memory (Nan et al., 2012; Bobby and Prakash, 2017), attention (Berger and Davelaar, 2018), and motor learning (Nan et al., 2020). To the best of our knowledge, there are no tACS multi-session studies investigating the session-to-session evolution of alpha dynamics during tACS online periods.

### Aftereffects and Their Potential Link With Brain Plasticity

So far, two major types of aftereffects have been considered to occur. The first is characterized by the same tendency of EEG behavior in the offline period relative to the online one, for example increased amplitude during a stimulation period (online period) and the persistence of increased amplitude in a poststimulation period (offline period). In both tACS and NFB, STA is considered to have a longer duration, ranging from minutes to hours (Ros et al., 2010; Ros and Gruzelier, 2011; Neuling et al., 2013; Kasten et al., 2016; Kasten and Herrmann, 2017). There is a great agreement in tACS as well as in NFB research indicating that this kind of aftereffect is the result of Hebbian plasticity (Ros et al., 2010, 2014a; Egner and Sterman, 2014; Veniero et al., 2015; Vossen et al., 2015; Kasten et al., 2016; Sitaram et al., 2017; Wischnewski and Schutter, 2017; Batail et al., 2019; Haberbosch et al., 2019) though only a couple of tACS and NFB studies have directly investigated the link between the EEG aftereffects and markers of Hebbian plasticity (Lee et al., 2019; Wischnewski et al., 2019; Markiewicz and Dobrowolska, 2020; Riddle et al., 2020a). The second type of aftereffect is characterized by opposite tendency in EEG behavior in the offline period relative to the online period, for example, decreased amplitude in the online period is followed by increased amplitude in the offline period. This kind of aftereffect has been shown to occur immediately after the termination of intervention by NFB or tACS (Garside et al., 2015; Nicholson et al., 2016; Deiber et al., 2020). Within the alpha-band research, OTA has been reported by several NFB studies (Kluetsch et al., 2014; Peeters et al., 2014; Nicholson et al., 2016; Ros et al., 2017a,b; Deiber et al., 2020) whereas the occurrence of OTA in tACS research is rather ambiguous (Gundlach et al., 2017; Haberbosch et al., 2019; Krawinkel et al., 2019; Zarubin et al., 2020). OTA has been attributed to the influence of homeostatic plasticity in both NFB (Kluetsch et al., 2014; Peeters et al., 2014; Nicholson et al., 2016; Ros et al., 2017a,b; Deiber et al., 2020) and tACS research (Garside et al., 2015; Gundlach et al., 2017; Ketz et al., 2018; Haberbosch et al., 2019; Zarubin et al., 2020). However, to the best to our knowledge, so far, neither tACS nor NFB studies have investigated a direct link between OTA and the neural markers specific for homeostatic plasticity.

However, it is necessary to highlight some limitations regarding a potential link between aftereffects and brain plasticity. First, the prevalence of the majority of our discussed NFB (Hanslmayr et al., 2005; Zoefel et al., 2011; Nan et al., 2012) and tACS studies reporting poststimulation alpha-band aftereffects used the alpha tACS protocols designed to stimulate parieto-occipital regions (Zaehle et al., 2010; Neuling et al., 2013; Helfrich et al., 2014b; Kasten et al., 2016; Kasten and Herrmann, 2017; Stecher et al., 2017; Berger et al., 2018; Ahn et al., 2019). On the other hand, alpha tACS was shown to be unsuccessful in inducing aftereffects in the alpha (mu) band after the stimulation of central brain regions (Gundlach et al., 2017). Based on these outcomes, it is possible that the dynamics of alpha aftereffects depend on the location of the stimulated brain areas. Another important point that needs to be mentioned is the potential dependence of aftereffects on the stimulated EEG frequency (Nowak et al., 2017; Guerra et al., 2019; Pozdniakov et al., 2021). Several studies report no aftereffects after beta and gamma tACS (Nowak et al., 2017; Guerra et al., 2019; Pozdniakov et al., 2021). According to the current body of knowledge, it appears that aftereffects are the most pronounced in the parieto-occipital alpha band. Nevertheless, in order to be able to deeply understand the aftereffects and their dynamics, future systematic research is necessary to uncover the dynamics of aftereffects in all EEG frequency bandwidths and also in various brain areas within one frequency bandwidth.

Based on the current literature, there are many tACS and NFB studies speaking in favor of the capability of these two neuromodulation methods to modulate alpha activity and improve brain functioning. However, it is necessary to say there are many factors, which have been found to influence the responsiveness of EEG activity to tACS and NFB. Consequently, these factors represent a source of noise to the interpretation of results of tACS and NFB studies. The following section is dedicated to the factors responsible for influencing brain responsiveness to tACS and NFB.

## Factors Influencing Responsiveness to tACS and NFB

In spite of many tACS and NFB studies, which have successfully modulated the target EEG activity, there is a potential to find the reports of NFB (Alkoby et al., 2018) and tACS participants (van Schouwenburg et al., 2018; Manzo et al., 2020; Ronconi et al., 2020) who showed no response to a particular neuromodulation method. A potential source of variability in response to both modalities has been attributed to different protocol designs, including parameters such as size and montages of electrodes, duration of intervention, and intensity and phase of tACS (Nitsche et al., 2015; Karabanov et al., 2016; Fertonani and Miniussi, 2017; Tavakoli and Yun, 2017; Alkoby et al., 2018). Also, anatomical and physiological specific features of a particular stimulated brain area were strongly proposed to play a very important role (Manzo et al., 2020). Another category of factors influencing brain responsiveness to external neuromodulation includes factors, which are connected to the brain states (Silvanto and Pascual-Leone, 2008; Paulus and Rothwell, 2016). In contrast to tACS research, it seems that NFB research has investigated more types of factors, which may influence brain responsiveness to NFB. Within tACS research, factors, such as aging (Fresnoza et al., 2020), actual health condition (Krause et al., 2014), baseline (pre-stimulation) level of the targeted EEG activity (Neuling et al., 2013; Alagapan et al., 2016; Ruhnau et al., 2016; Bächinger et al., 2017; Lefebvre et al., 2017; Berger et al., 2018), placebo (Antal and Herrmann, 2016), ceiling effect (Krause et al., 2014; Fresnoza et al., 2020), a specific type of cognitive activity during which tACS is induced (Feurra et al., 2013), and illumination condition (Kanai et al., 2008, 2010; Stecher et al., 2017), have been examined. In NFB research, several factors related to brain state have been investigated, including baseline level of the target EEG activity (Travis et al., 1974; Wan et al., 2014; Nicholson et al., 2016; Nan et al., 2020), the level of illumination of the treatment room (Paskewitz and Orne, 1973; Cram et al., 1977), ceiling effect (Hardt and Kamiya, 1978), placebo (Mullinix et al., 1978; Plotkin and Rice, 1981; Holroyd et al., 1984; Kotchoubey et al., 2001; Thibault and Raz, 2017; Shibata et al., 2019), aging (Staufenbiel et al., 2014), the brain morphology (Enriquez-Geppert et al., 2013), healthy brain vs. pathological condition (Ros et al., 2017a), the effect of fatigue (Choobforoushzadeh et al., 2015), the effect of anxiety (Hardt and Kamiya, 1978; Kadosh and Staunton, 2019), the effect of mental strategies used for enhancing NFB-rewarded neural activity (Lacroix and Roberts, 1978; Sepulveda et al., 2016; Lubianiker et al., 2019; Shibata et al., 2019), the effects of mood (Kadosh and Staunton, 2019), and the personality traits (Ancoli and Kamiya, 1978; Peciuliene et al., 2015). Among all these investigated domains, both tACS and NFB have investigated the following four types of factor in relation to the responsiveness of alpha band to neuromodulation: (1) baseline level of targeted EEG activity, (2) illumination condition, (3) placebo effect, and (4) ceiling effect. A comparative study of these mentioned variability factors for tACS and NFB might represent fruitful future research. Nevertheless, it is necessary to bear in mind that the interaction between tACS and state-dependent factors is completely different from the interaction between NFB and state-dependent factors in the following way: the interaction between tACS and state-dependent factors represents the interaction between exogenous electric fields (tACS) and endogenous ones (neuronal activity) (Reato et al., 2013; Paulus and Rothwell, 2016). On the other hand, the interaction between NFB and state-dependent factors represents an interplay between the sources of the endogenous activity responsible for regulating neural activity toward NFB-rewarded patterns and the sources of endogenous activity modulated by state-dependent factors (Kadosh and Staunton, 2019).

## Conclusions and Future Directions

The aim of this review was to compare the current state of knowledge regarding tACS and NFB in connection with their effects on the online and offline alpha band, the underlying mechanisms of tACS and NFB responsible for the modulation of the online and the offline EEG activity, dynamics, duration, and potential benefits of alpha-band aftereffects, and the factors responsible for variability in response to tACS and NFB.

Starting with online effects, single-session tACS and NFB studies have reported that the particular neuromodulation methods can affect various alpha-band properties, including changes in amplitude, coherence, frequency, and CFIs. In the multi-session studies, there are many NFB studies, which scrutinized the online dynamics of the alpha band whereas, best to our knowledge, no multi-session tACS study investigating online EEG has been done. We believe it is worth investigating the mechanisms responsible for the magnitude of responsiveness of online EEG to multi-session tACS and the mechanisms responsible for the magnitude of that kind of responsiveness. In relation to the mechanisms responsible for the modulation of online of EEG activity, different mechanisms are considered for tACS and NFB.

Both NFB and tACS studies report that alpha aftereffects can be linked to improved brain functioning. The duration of the alpha aftereffect is comparable for tACS and NFB including single- and multi-session studies.

Hebbian plasticity is considered by tACS as well as by NFB to be responsible for EEG aftereffects which display the same behavior as online EEG activity. Homeostatic plasticity is considered by both tACS and NFB to be responsible for the occurrence of EEG aftereffects which display the opposite behavior as online EEG activity. However, just a couple of tACS and NFB studies have investigated a direct link between aftereffects and the neural markers of Hebbian plasticity. To the best of our knowledge, a direct link between aftereffects and the markers of homeostatic plasticity has been investigated by neither tACS nor NFB study. We believe that studying a link between brain plasticity and the dynamics of aftereffects represents an interesting and a fruitful research field.

Transcranial alternating current stimulation- and NFB-induced aftereffects are usually studied in connection with the amplitude and coherence of the alpha band. We suggest it might be interesting to study the dynamics, the duration of persistence, and the potential behavioral benefits of tACS- and NFB-induced aftereffects in the frequency dynamics of the targeted EEG band and in its CFIs. We also propose that it would be fruitful to systematically study aftereffects in various frequency bandwidths and compare their behavior between tACS and NFB.

The final domain of our interest in this article was dedicated to discussing and comparing the factors responsible for variability in responsiveness to tACS and NFB. In contrast to tACS, significantly more factors have been addressed in NFB studies so far. Interestingly, we have found four common factors, which were well-studied in tACS and in NFB experiments: level of baseline (pre-stimulation) activity, level of illumination of the treatment room, the placebo effect, and the ceiling effect. Regarding the factors investigated for NFB but not yet addressed by tACS research such as level of baseline anxiety and personality trait, we believe that it may be worthwhile to determine whether and how these factors affect responsiveness to tACS. Also, it might be interesting to compare the magnitude of the effects of all aforementioned state-dependent factors on electrophysiological and behavioral responses to tACS and NFB. We believe that such types of future studies will expand our understanding of the mechanisms of brain responsiveness to tACS and NFB. Hopefully, the results of the future studies will lead to a better determination of which experimental and clinical situations are best suited for tACS and which ones are best suited for NFB.

## Author Contributions

MO wrote the preliminary and final manuscript. EK helped with formal editing and with administration issues. Both authors contributed to the article and approved the submitted version.

## Conflict of Interest

The authors declare that the research was conducted in the absence of any commercial or financial relationships that could be construed as a potential conflict of interest.
